# A DNA Barcode Reference Library for Trichoptera of Jingpo Lake: Taxonomic Diversity and Molecular Identification Basics

**DOI:** 10.3390/insects17080857

**Published:** 2026-08-17

**Authors:** Tongyin Xie, Lu Chai, Ni Zhang, Na Wang, Xinyu Ge, Chuncai Yan

**Affiliations:** 1 College of Plant Protection, Northeast Agricultural University, Harbin 150030, China; xietongyin@163.com; 2 Tianjin Key Laboratory of Conservation and Utilization of Animal Diversity, College of Life Sciences, Tianjin Normal University, Tianjin 300387, China; 2410170016@stu.tjnu.edu.cn (L.C.); 2510170030@stu.tjnu.edu.cn (N.Z.); 2510170025@stu.tjnu.edu.cn (N.W.)

**Keywords:** Trichoptera, DNA barcoding, COI, freshwater biodiversity, new record

## Abstract

Jingpo Lake in Northeast China is a vital aquatic ecosystem, yet it lacks baseline molecular data for its insects. Trichoptera (caddisflies) are excellent ecological bioindicators, but their accurate identification is often hindered by morphological similarities, life-stage limitations, and insufficient DNA barcode records. To address this gap, this study combined morphological identification with DNA barcoding to establish a local reference database. From 285 collected adult caddisflies, 105 representative specimens were selected for genetic analysis. The survey identified 22 species across 9 families, revealing four new records for China and six for Heilongjiang Province. Analyses confirmed the high efficacy of the COI marker, demonstrating low intraspecific genetic divergence (0.78%) and high interspecific divergence (17.80%), with a clear barcode gap separating all examined species. Species accumulation curves indicated robust sampling of the main genera, although expanded collection efforts could still uncover additional species. In conclusion, this study provides a valuable DNA barcode reference library for Trichoptera in the Jingpo Lake region. It establishes foundational data for regional biodiversity inventories and future ecological monitoring based on environmental DNA (eDNA) or metabarcoding approaches.

## Simple Summary

Aquatic insects are useful indicators for evaluating the condition of aquatic ecosystems, but reliable species identification is often limited by incomplete regional reference data. In this study, Trichoptera (caddisflies) were surveyed in the Jingpo Lake area of Northeast China, and mitochondrial COI barcodes were generated for representative specimens covering all species collected during the survey. The resulting dataset included 105 COI barcodes from 22 species, 14 genera, and 9 families. COI barcodes showed clear separation among species and were generally consistent with morphological identification. The results provide a local molecular reference for Trichoptera in Jingpo Lake and will support future biodiversity assessment, environmental DNA monitoring, and freshwater ecosystem conservation in the region.

## 1. Introduction

Freshwater ecosystems are a core component of Earth’s biosphere, serving as a critical nexus for material cycling and energy flow between terrestrial and aquatic ecosystems [1]. Although they cover approximately 0.8% of the Earth’s total surface area, freshwater ecosystems support more than 10% of the world’s described species, establishing them as pivotal global hotspots for biodiversity conservation [2]. These ecosystems deliver a multidimensional suite of critical ecosystem services, encompassing resource provisioning, ecological regulation, and habitat support [3]. Currently, freshwater ecosystems worldwide are facing an unprecedented degradation crisis. The rate of decline in freshwater biodiversity far outpaces that of terrestrial and marine ecosystems, emerging as one of the most formidable challenges in the field of global biodiversity conservation [4]. The 2019 IPBES Global Assessment Report explicitly notes that nearly one million animal and plant species worldwide are threatened with extinction, with freshwater species exhibiting the steepest population declines [5].

Over the past 50 years, global freshwater species populations have declined by more than 80% in abundance—a decline far steeper than that observed in marine and terrestrial species [6]. The rapid degradation of freshwater ecosystems and concomitant loss of biodiversity stem from the cumulative impacts of multiple anthropogenic and environmental stressors, with primary drivers including overexploitation of water resources, watershed pollution, biological invasions, and climate change-induced hydrological regime alterations [7]. Of these stressors, the synergistic effects of climate change and intensifying anthropogenic activities are profoundly reshaping the hydrological regimes of global water bodies, which has emerged as a dominant driver of the ongoing global freshwater biodiversity decline [8,9].

Against this global backdrop of accelerating freshwater ecosystem degradation, lakes emerge as particularly vulnerable and ecologically significant systems that warrant focused research attention. As a core component of the terrestrial hydrosphere, lakes are exceptionally sensitive to disturbances from climate change and anthropogenic activities. They function as critical sentinels that integrate signals of global change drivers and regional ecological responses, and represent central repositories of freshwater biodiversity and ecosystem services in China. Lake ecosystems deliver indispensable ecosystem services spanning hydrological regulation, biodiversity conservation, freshwater provision, and socioeconomic support for fisheries, agriculture, navigation, and tourism [10]. Recent authoritative surveys show China contains 2693 natural lakes (excluding ephemeral playas) covering 81,414.6 km^2^, roughly 0.9% of the country’s total land area [11].

Jingpo Lake, located in Mudanjiang City, Heilongjiang Province, Northeast China, spans the geographic coordinates 43°30′–44°20′ N and 128°07′–129°06′ E. It has a surface area of 90.3 km^2^, a mean water depth of 40 m, a maximum water depth of 65 m, and a water storage capacity ranging from 9 × 10^8^ to 16 × 10^8^ m^3^ [12]. It is the largest alpine lava-dammed lake by area in China and one of China’s 27 national volcanic geoparks [13]. As a critical freshwater ecosystem in Northeast China, Jingpo Lake combines exceptional ecological value with vital socioeconomic functions and livelihood security roles. Within the regional ecological security framework, it functions as a key ecological barrier for Northeast China, fulfilling the core ecological role of sustaining aquatic biodiversity and providing irreplaceable habitats for the survival and reproduction of rare endemic aquatic flora and fauna in the region.

Over the past several decades, accelerating industrialization, urbanization, agricultural intensification, and tourism expansion in the Jingpo Lake Basin have drastically amplified anthropogenic pressures, triggering severe cumulative environmental degradation including eutrophication, excessive pollutant loading, and precipitous declines in aquatic biodiversity [14]. Water quality in Jingpo Lake degraded from Class I to Class IV according to the Chinese Environmental Quality Standards for Surface Water (GB 3838-2002) between the mid-20th century and 2018, marking a severe and sustained deterioration of the aquatic environment [15]. While recent large-scale, basin-wide aquatic ecological restoration projects have successfully restored water quality to Class III, aquatic ecosystem recovery is inherently a protracted process. Thus, the rebound of biodiversity and reconstruction of ecosystem functions depend critically on long-term, multidimensional biological monitoring and systematic ecological assessment [16].

**Figure 1:**
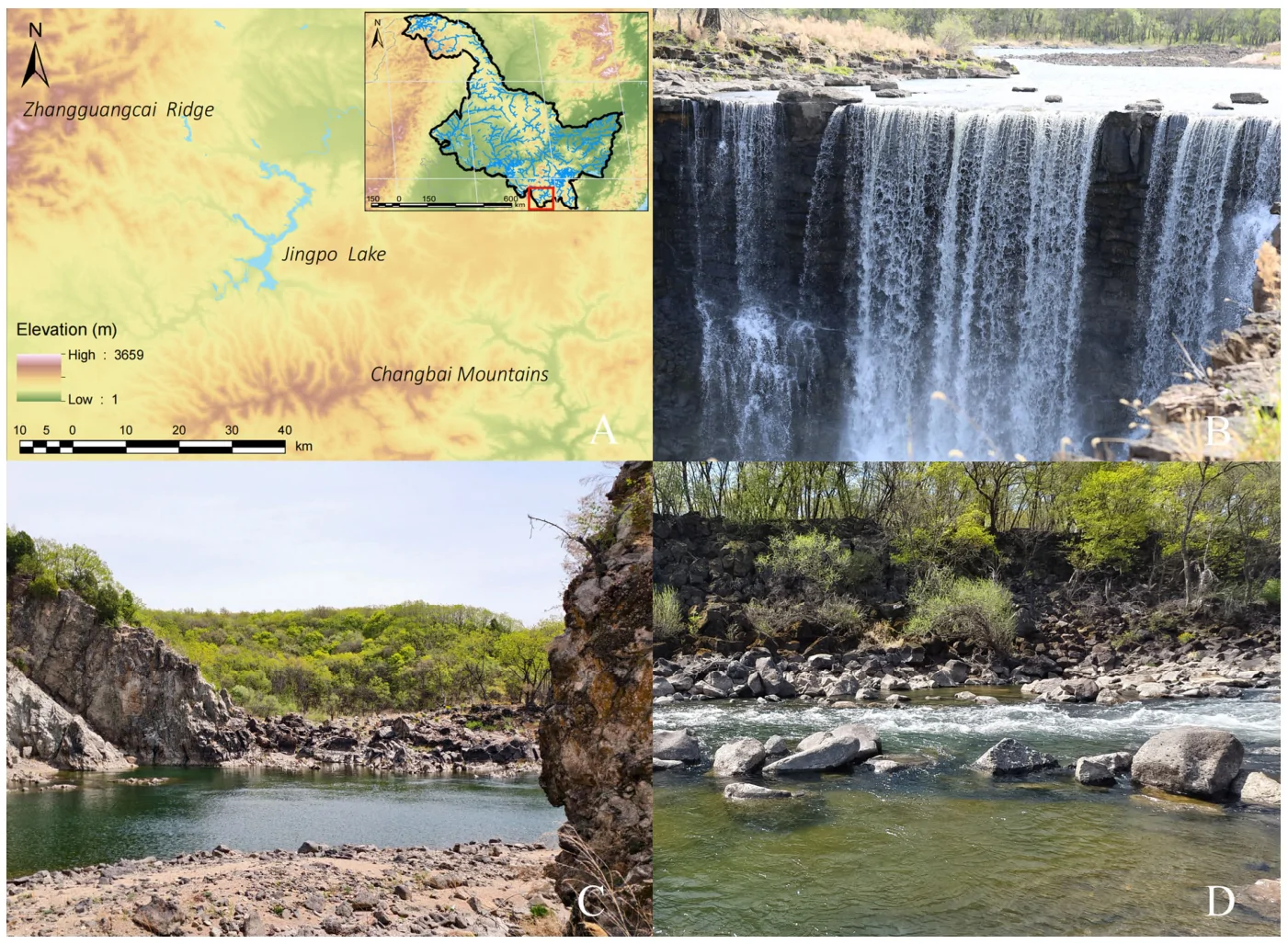
Location of Jingpo Lake and representative sampling habitats of Trichoptera. (**A**) Location of Jingpo Lake in China; (**B**–**D**) representative sampling habitats, showing rocky stream channels, waterfall-associated habitats, lake-shore environments, shallow running water, exposed stones, and coarse substrates.

As a representative freshwater ecosystem, Jingpo Lake supports exceptionally diverse aquatic invertebrate communities. These organisms form the core of the lake’s food web structure and serve as sensitive bioindicators of aquatic ecosystem health [17,18]. Trichoptera (caddisflies) are among the most ecologically significant groups within this assemblage (Figure 1). As the seventh largest order of Insecta, Trichoptera are characterized by predominantly aquatic larvae that secrete silk filaments to build fixed retreats or portable cases. This unique life history trait enables them to perform irreplaceable roles in material cycling and energy flow within freshwater ecosystems, particularly in lotic (stream) environments [19]. However, taxonomic and diversity studies on Trichoptera in the basins of Heilongjiang Province are still relatively scarce at present, with a severe lack of baseline species data. Meanwhile, traditional morphology-based species identification methods have extremely high requirements for taxonomic expertise, which not only fail to meet the demands of large-scale and high-frequency aquatic ecological monitoring, but also are highly prone to species misidentification or oversight of cryptic species.

DNA barcoding technology enables rapid and accurate species identification through a short, standardized gene fragment, effectively breaking through the technical bottleneck of traditional morphological taxonomy [20]. This technology allows for rapid adult-larva association of species, and possesses multiple key advantages including precise species identification, efficient detection of cryptic species, and standardized data comparison [21,22,23]. In recent years, the integrated application of environmental DNA (eDNA) technology and DNA barcoding technology has provided a novel technical pathway for biodiversity monitoring in freshwater ecosystems [17]. eDNA technology achieves non-invasive, rapid qualitative species detection by detecting extracellular genetic material released by organisms into environmental matrices (e.g., water samples), and is particularly suitable for early warning of biological invasions and large-scale basin biodiversity surveys [24,25,26]. When eDNA metabarcoding analysis is coupled with a high-quality, full-coverage species DNA barcode reference library, accurate species identification can be accomplished without collecting large numbers of physical biological specimens, enabling rapid evaluation of the effectiveness of aquatic ecosystem restoration [27].

However, the large-scale application of eDNA technology in freshwater biodiversity monitoring remains hindered by a critical bottleneck: the incompleteness of DNA barcode reference libraries [28,29]. While global DNA barcoding studies on Trichoptera have advanced considerably, barcode sequence coverage for Trichoptera species in Asia—particularly Northeast China—remains drastically limited, leaving most native regional species without standardized reference barcode data [30]. This paucity of geographically targeted reference data directly impairs eDNA detection performance, manifesting as reduced sequence-matching efficiency and compromised species identification accuracy [31,32]. Accordingly, the construction of high-quality, localized DNA barcode reference libraries for specific study regions is a fundamental prerequisite for ensuring the accuracy and reliability of eDNA biomonitoring results [33].

In this study, we adopted an integrated research approach combining traditional morphological taxonomy and molecular biological techniques to systematically investigate the species composition and faunal characteristics of Trichoptera in the Jingpo Lake Basin, and constructed a comprehensive, accurate, and localized DNA barcode reference library for Trichoptera taxa in this water body. Our findings enhance the accuracy and efficiency of local Trichoptera species identification and provide a robust scientific foundation for evaluating the aquatic ecosystem restoration effectiveness of Jingpo Lake and formulating targeted biodiversity conservation strategies. Furthermore, this study fills a critical regional data gap in Trichoptera DNA barcoding for Northeast China, and offers essential baseline data and theoretical support for global freshwater ecosystem conservation initiatives and international data sharing efforts.

## 2. Materials and Methods

### 2.1. Sample Collection and Morphological Identification

From 6 to 7 August 2025, a field survey of Trichoptera diversity was conducted in Jingpo Lake National Scenic Area, Heilongjiang Province, China. Specimens were collected by light trapping from dusk until midnight, with a white cotton sheet hung vertically in front of the light source to maximize trapping efficiency [34]. All sampling sites were situated within the Jingpo Lake National Scenic Area and primarily included lakeshore habitats, inflowing tributaries, and adjacent moist riparian zones. All captured specimens were immediately fixed in 85% ethanol in the field to minimize tissue degradation; upon return to the laboratory, they were stored long-term at −20 °C prior to genomic DNA extraction [35].

Species-level morphological identification was conducted primarily by examining male genitalic structures, with supporting characters including wing venation details and other external morphological features. The male genital segments were removed and cleared by boiling in 10% KOH for 5–15 min, rinsed in distilled water, and subsequently examined and illustrated in glycerin [36]. All identifications were verified against published regional taxonomic keys and authoritative monographs for Palaearctic Trichoptera [37]. Specimen dissection, observation, and image acquisition were performed under a Leica M205 C stereomicroscope (Leica Microsystems GmbH, Wetzlar, Germany) equipped with a digital imaging system. All voucher specimens are deposited in the specimen collection of the College of Life Sciences, Tianjin Normal University.

### 2.2. Molecular Experiments

To clarify the genetic diversity of the trichopteran species in this region, 105 adult specimens representing all species recovered during the survey were selected for COI barcode sequencing and analysis. Genomic DNA was extracted from the collected adult specimens. To preserve the external morphology of voucher specimens as far as possible, one hind leg was removed from each individual for DNA extraction, while the remaining body was retained for subsequent morphological verification. Genomic DNA was extracted using the TIANamp Genomic DNA Kit for animal tissues (Tiangen Biotech Co., Ltd., Beijing 100192, China; Cat. No. DP304-03), following the manufacturer’s protocol.

PCR amplification was performed using the metazoan universal primers LCO1490 and HCO2198 [38]. Each PCR reaction had a total volume of 50 μL and contained 25 μL of 2 × Taq PCR Master Mix II (Dye Plus) (Vazyme, Nanjing, China), 2 μL of DNA template, 2 μL of each forward and reverse primer, and ddH_2_O to 50 μL [22]. The PCR cycling conditions consisted of an initial denaturation at 94 °C for 1 min, followed by 5 cycles of denaturation at 94 °C for 1 min, annealing at 45 °C for 1.5 min, and extension at 72 °C for 1.5 min; this was followed by 35 cycles of denaturation at 94 °C for 1 min, annealing at 52 °C for 1.5 min, and extension at 72 °C for 1 min. A final extension was performed at 72 °C for 5 min, followed by holding at 4 °C. Amplification products were examined by electrophoresis on 1% agarose gels. Products with target bands were subjected to bidirectional Sanger sequencing by Beijing Genomics Institute (BGI, Beijing 518083, China), yielding the final COI barcodes.

### 2.3. Sequence Processing and DNA Barcode Analyses

A total of 105 COI sequences were obtained, and the sequences together with associated specimen metadata were uploaded to the BOLD Systems platform under the dataset title “Barcoding Study of Trichoptera in Jingpo Lake, Heilongjiang Province, China”. Raw bidirectional chromatograms were assembled, trimmed, and quality-checked in Geneious Prime v2024.0.5 [39]. After manual verification, sequences were aligned in MEGA 11 using MUSCLE alignment [40]. The aligned sequences were translated into amino acid sequences to screen for stop codons, frameshift mutations, and obvious sequencing errors, thereby ensuring sequence reliability.

Phylogenetic relationships were reconstructed in MEGA 11 using the neighbor-joining (NJ) method [41]. Genetic distances were calculated using the Kimura two-parameter (K2P) model [42], and nodal support was assessed with 1000 bootstrap replicates. Missing positions and ambiguous bases were treated as missing data, and gaps were handled using pairwise deletion. Nodes with bootstrap support greater than 50% were displayed in the final tree. The tree file was subsequently imported into iTOL v7 for visual editing [43].

Genetic distances, barcode gaps, and species accumulation curves were analyzed using the BOLD workbench [44]. Distance Summary and Barcode Gap Analysis were conducted based on the standard COI barcode, with a minimum sequence length threshold of 200 bp; gaps and ambiguous bases were handled by pairwise deletion. Genetic distances were summarized at the intraspecific, interspecific-within-genus, and intergeneric-within-family levels, and the minimum, maximum, mean, and standard error were reported. Barcode gap analysis was used to evaluate the discriminatory power of COI barcodes for species identification by comparing the maximum intraspecific genetic distance of each species with the minimum interspecific distance to its nearest-neighbor species.

Species accumulation curves were generated at both the species and genus levels, using family as the grouping unit. The K2P distance model was applied with 20 iterations, while all other parameters were kept at their default settings. This analysis was used to assess sampling sufficiency, potential species richness, and priority directions for further sampling among different families.

## 3. Results

### 3.1. Taxonomic Diversity

A total of 285 adult caddisfly specimens were collected during the survey. Based on morphological examination, these specimens were assigned to 22 species in 14 genera and 9 families. The species list is as follows:


 


**Suborder Annulipalpia Martynov, 1924**

**Family Ecnomidae Ulmer, 1903**

**Genus *Ecnomus* McLachlan, 1864**

**Species *Ecnomus tenellus* Rambur, 1842** (first record for Heilongjiang Province)

**Material examined**: 31 ♂, China, Heilongjiang Province, Ning’an City, Bohai Town, Jingpo Lake National Scenic Area, 128°56′7.21″ N, 44°2′30.08″ E, alt. 379 m, 6 August 2025, light trap, leg. Zhichao Zhang (Z.C. Zhang); 21 ♂, China, Heilongjiang Province, Ning’an City, Bohai Town, Jingpo Lake National Scenic Area, 128°57′0.07″ N, 44°3′18.30″ E, alt. 366 m, 7 August 2025, light trap, leg. Z.C. Zhang; 51 ♂, China, Heilongjiang Province, Ning’an City, Bohai Town, Jingpo Lake National Scenic Area, 128°57′0.01″ N, 44°3′18.30″ E, alt. 366 m, 7 August 2025, light trap, leg. Z.C. Zhang.

**Distribution**: China (Guangdong, Anhui, Jiangxi, Jiangsu, Hubei, Sichuan, Yunnan, Tibet, Taiwan, Heilongjiang).


 


**Species *Ecnomus yamashironis* Tsuda, 1942** (first record for Heilongjiang Province)

**Material examined**: 1 ♂, China, Heilongjiang Province, Ning’an City, Bohai Town, Jingpo Lake National Scenic Area, 128°56′7.21″ N, 44°2′30.08″ E, alt. 379 m, 6 August 2025, light trap, leg. Z.C. Zhang; 1 ♂, China, Heilongjiang Province, Ning’an City, Bohai Town, Jingpo Lake National Scenic Area, 128°57′0.00″ N, 44°3′18.30″ E, alt. 366 m, 7 August 2025, light trap, leg. Z.C. Zhang.

**Distribution**: China (Hubei, Jiangxi, Heilongjiang).


 


**Family Hydropsychidae Curtis, 1835**

**Genus *Arctopsyche* McLachlan, 1868**

**Species *Arctopsyche palpata* Martynov, 1934**

**Material examined**: 2 ♂, China, Heilongjiang Province, Ning’an City, Bohai Town, Jingpo Lake National Scenic Area, 128°56′7.21″ N, 44°2′30.08″ E, alt. 379 m, 6 August 2025, light trap, leg. Z.C. Zhang.

**Distribution**: China (Jilin, Heilongjiang, Inner Mongolia).


 


**Genus *Cheumatopsyche* Wallengren, 1891**

**Figure 2:**
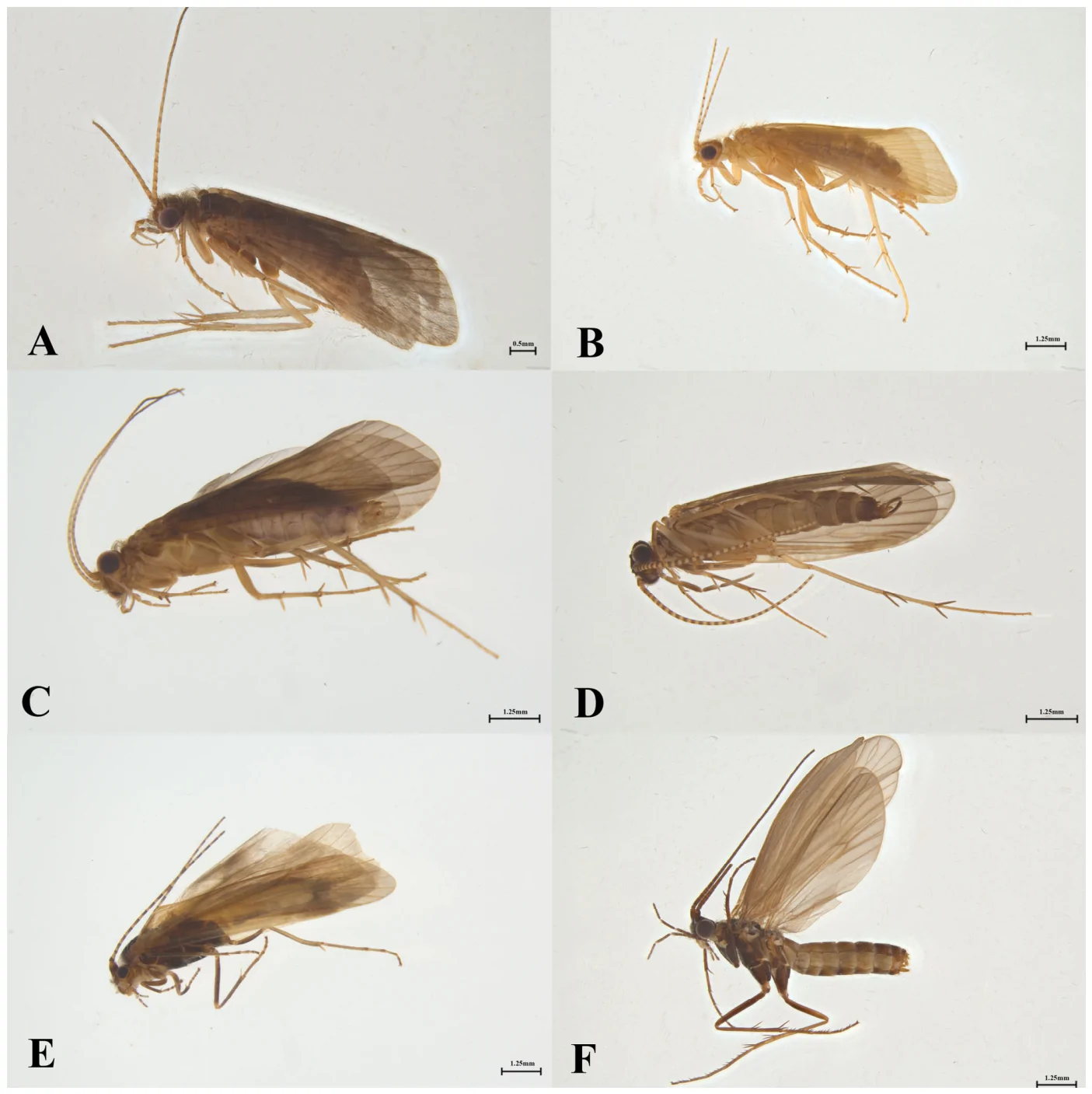
Photographs of trichopteran species recorded from Jingpo Lake. (**A**) *Cheumatopsyche brevilineata*; (**B**) *Hydropsyche kozhantschikovi*; (**C**) *Hydropsyche orientalis*; (**D**) *Kisaura aurascens*; (**E**) *Ceraclea sibirica*; (**F**) *Molannodes tinctus*.

**Species *Cheumatopsyche brevilineata* Iwata, 1927** (first record for Heilongjiang Province; Figure 2A)

**Material examined**: 14 ♂, China, Heilongjiang Province, Ning’an City, Bohai Town, Jingpo Lake National Scenic Area, 128°56′7.21″ N, 44°2′30.08″ E, alt. 379 m, 6 August 2025, light trap, leg. Z.C. Zhang.

**Distribution**: China (Beijing, Tianjin, Taiwan, Heilongjiang).


 


**Genus *Hydropsyche* Pictet, 1834**

**Species *Hydropsyche kozhantschikovi* Martynov, 1924** (Figure 2B)

**Material examined**: 1 ♂, China, Heilongjiang Province, Ning’an City, Bohai Town, Jingpo Lake National Scenic Area, 128°57′0.06″ N, 44°3′18.30″ E, alt. 366 m, 7 August 2025, light trap, leg. Z.C. Zhang; 3 ♂, China, Heilongjiang Province, Ning’an City, Bohai Town, beside waterfall of Jingpo Lake National Scenic Area, 128°57′0.00″ N, 44°3′18.30″ E, alt. 366 m, 7 August 2025, light trap, leg. Z.C. Zhang.

**Distribution**: China (Heilongjiang, Beijing).


 


**Species *Hydropsyche orientalis* Martynov, 1934** (Figure 2C)

**Material examined**: 8 ♂, China, Heilongjiang Province, Ning’an City, Bohai Town, Jingpo Lake National Scenic Area, 128°56′7.21″ N, 44°2′30.08″ E, alt. 379 m, 6 August 2025, light trap, leg. Z.C. Zhang.

**Distribution**: China (Shanxi; Heilongjiang, Jilin, Beijing, Xinjiang).


 


**Species *Hydropsyche valvata* Martynov, 1927**

**Material examined**: 1 ♂, China, Heilongjiang Province, Ning’an City, Bohai Town, Jingpo Lake National Scenic Area, 128°57′0.06″ N, 44°3′18.30″ E, alt. 366 m, 7 August 2025, light trap, leg. Z.C. Zhang.

**Distribution**: China (Heilongjiang, Shanxi, Yunnan, Zhejiang, Hubei, Anhui).


 


**Genus *Macrostemum* Kolenati, 1859**

**Species *Macrostemum radiatum* McLachlan, 1872** (first record for Heilongjiang Province)

**Material examined**: 3 ♂, China, Heilongjiang Province, Ning’an City, Bohai Town, Jingpo Lake National Scenic Area, 128°56′7.21″ N, 44°2′30.08″ E, alt. 379 m, 6 August 2025, light trap, leg. Z.C. Zhang; 10 ♂, China, Heilongjiang Province, Ning’an City, Bohai Town, Jingpo Lake National Scenic Area, 128°57′0.06″ N, 44°3′18.30″ E, alt. 366 m, 7 August 2025, light trap, leg. Z.C. Zhang; 6 ♂, China, Heilongjiang Province, Ning’an City, Bohai Town, Jingpo Lake National Scenic Area, 128°57′0.01″ N, 44°3′18.30″ E, alt. 366 m, 7 August 2025, light trap, leg. Z.C. Zhang.

**Distribution**: China (Anhui, Fujian, Zhejiang, Guangxi, Jilin, Jiangsu, Jiangxi, Xinjiang, Heilongjiang).


 


**Family Philopotamidae Stephens, 1829**

**Genus *Kisaura* Ross, 1956**

**Species *Kisaura aurascens* Martynov, 1934** (first record for China; Figure 2D)

**Material examined**: 6 ♂, China, Heilongjiang Province, Ning’an City, Bohai Town, Jingpo Lake National Scenic Area, 128°56′7.21″ N, 44°2′30.08″ E, alt. 379 m, 6 August 2025, light trap, leg. Z.C. Zhang.

**Distribution**: China (Heilongjiang); Japan [45]; Russia [46]; North Korea [47]; South Korea [48].


 


**Suborder Integripalpia Martynov, 1924**

**Family Lepidostomatidae Ulmer, 1903**

**Genus *Lepidostoma* Rambur, 1842**

**Species *Lepidostoma albardanum* Ulmer, 1906**

**Material examined**: 2 ♂, China, Heilongjiang Province, Ning’an City, Bohai Town, Jingpo Lake National Scenic Area, 128°56′7.21″ N, 44°2′30.08″ E, alt. 379 m, 6 August 2025, light trap, leg. Z.C. Zhang.

**Distribution**: China (Heilongjiang, Jilin).


 


**Species *Lepidostoma elongatum* Martynov, 1935**

**Material examined**: 6 ♂, China, Heilongjiang Province, Ning’an City, Bohai Town, Jingpo Lake National Scenic Area, 128°56′7.21″ N, 44°2′30.08″ E, alt. 379 m, 6 August 2025, light trap, leg. Z.C. Zhang.

**Distribution**: China (Heilongjiang, Jilin).


 


**Family Leptoceridae Leach, 1815**

**Genus *Ceraclea* Stephens, 1829**

**Species *Ceraclea albimacula* Rambur, 1842**

**Material examined**: 51 ♂, China, Heilongjiang Province, Ning’an City, Bohai Town, Jingpo Lake National Scenic Area, 128°56′7.21″ N, 44°2′30.08″ E, alt. 379 m, 6 August 2025, light trap, leg. Z.C. Zhang; 1 ♂, China, Heilongjiang Province, Ning’an City, Bohai Town, Jingpo Lake National Scenic Area, 128°57′0.06″ N, 44°3′18.30″ E, alt. 366 m, 7 August 2025, light trap, leg. Z.C. Zhang; 1 ♂, China, Heilongjiang Province, Ning’an City, Bohai Town, Jingpo Lake National Scenic Area, beneath Diaoshuilou Waterfall, 128°56′52.53″ N, 44°2′46.72″ E, alt. 366 m, 7 August 2025, light trap, leg. Z.C. Zhang; 3 ♂, China, Heilongjiang Province, Ning’an City, Bohai Town, Jingpo Lake National Scenic Area, 128°57′0.01″ N, 44°3′18.30″ E, alt. 366 m, 7 August 2025, light trap, leg. Z.C. Zhang.

**Distribution**: China (Heilongjiang).


 


**Species *Ceraclea lobulata* Martynov, 1935**

**Material examined**: 21 ♂, China, Heilongjiang Province, Ning’an City, Bohai Town, Jingpo Lake National Scenic Area, 128°56′7.21″ N, 44°2′30.08″ E, alt. 379 m, 6 August 2025, light trap, leg. Z.C. Zhang; 13 ♂, China, Heilongjiang Province, Ning’an City, Bohai Town, Jingpo Lake National Scenic Area, 128°57′0.06″ N, 44°3′18.30″ E, alt. 366 m, 7 August 2025, light trap, leg. Z.C. Zhang; 4 ♂, China, Heilongjiang Province, Ning’an City, Bohai Town, Jingpo Lake National Scenic Area, 128°57′0.01″ N, 44°3′18.30″ E, alt. 366 m, 7 August 2025, light trap, leg. Z.C. Zhang.

**Distribution**: China (Heilongjiang).


 


**Species *Ceraclea sibirica* Ulmer, 1906** (first record for China; Figure 2E)

**Material examined**: 1 ♂, China, Heilongjiang Province, Ning’an City, Bohai Town, Jingpo Lake National Scenic Area, 128°56′7.21″ N, 44°2′30.08″ E, alt. 379 m, 6 August 2025, light trap, leg. Z.C. Zhang.

**Distribution**: China (Heilongjiang); South Korea [49]; Russia [50]; North Korea [47]; Mongolia [51].


 


**Genus *Oecetis* McLachlan, 1877**

**Species *Oecetis brachyura* Yang & Morse, 1997** (first record for Heilongjiang Province)

**Material examined**: 1 ♂, China, Heilongjiang Province, Ning’an City, Bohai Town, Jingpo Lake National Scenic Area, 128°56′7.21″ N, 44°2′30.08″ E, alt. 379 m, 6 August 2025, light trap, leg. Z.C. Zhang.

**Distribution**: China (Guizhou, Heilongjiang).


 


**Species *Oecetis bullata* Yang & Morse, 1997** (first record for Heilongjiang Province)

**Material examined**: 1 ♂, China, Heilongjiang Province, Ning’an City, Bohai Town, Jingpo Lake National Scenic Area, 128°56′7.21″ N, 44°2′30.08″ E, alt. 379 m, 6 August 2025, light trap, leg. Z.C. Zhang.

**Distribution**: China (Anhui, Heilongjiang).


 


**Family Limnephilidae Kolenati, 1848**

**Genus *Hydatophylax* Wallengren, 1891**

**Species *Hydatophylax soldatovi* Martynov, 1914**

**Material examined**: 7 ♂, China, Heilongjiang Province, Ning’an City, Bohai Town, Jingpo Lake National Scenic Area, 128°56′7.21″ N, 44°2′30.08″ E, alt. 379 m, 6 August 2025, light trap, leg. Z.C. Zhang.

**Distribution**: China (Jilin, Heilongjiang).


 


**Family Molannidae Wallengren, 1891**

**Genus *Molanna* Curtis, 1834**

**Species *Molanna moesta* Banks, 1906**

**Material examined**: 1 ♂, China, Heilongjiang Province, Ning’an City, Bohai Town, Jingpo Lake National Scenic Area, 128°56′7.21″ N, 44°2′30.08″ E, alt. 379 m, 6 August 2025, light trap, leg. Z.C. Zhang; 1 ♂, China, Heilongjiang Province, Ning’an City, Bohai Town, Jingpo Lake National Scenic Area, 128°57′0.06″ N, 44°3′18.30″ E, alt. 366 m, 7 August 2025, light trap, leg. Z.C. Zhang.

**Distribution**: China (Heilongjiang, Jiangxi, Guangdong, Sichuan, Guizhou, Yunnan).


 


**Genus *Molannodes* McLachlan, 1866**

**Species *Molannodes tinctus* McLachlan, 1866** (first record for China; Figure 2F)

**Material examined**: 1 ♂, China, Heilongjiang Province, Ning’an City, Bohai Town, Jingpo Lake National Scenic Area, 128°57′0.01″, 44°3′18.30″, alt. 366 m, 7 August 2025, light trap, leg. Z.C. Zhang.

**Distribution**: China (Heilongjiang); Germany [52]; Russia [53]; France [54]; Mongolia [46]; Ukraine [55]; Czech Republic [56]; Belgium [57]; United States [58]; Kazakhstan [59]; Norway [60]; Finland [61].


 


**Family Rhyacophilidae Stephens, 1836**

**Genus *Rhyacophila* Pictet, 1834**

**Species *Rhyacophila angulata* Martynov, 1910**

**Material examined**: 3 ♂, China, Heilongjiang Province, Ning’an City, Bohai Town, Jingpo Lake National Scenic Area, 128°57′0.06″ N, 44°3′18.30″ E, alt. 366 m, 7 August 2025, light trap, leg. Z.C. Zhang; 5 ♂, China, Heilongjiang Province, Ning’an City, Bohai Town, Jingpo Lake National Scenic Area, 128°57′0.01″ N, 44°3′18.30″ E, alt. 366 m, 7 August 2025, light trap, leg. Z.C. Zhang.

**Distribution**: China (Heilongjiang).


 


**Species *Rhyacophila retracta* Martynov, 1914**

**Material examined**: 2 ♂, China, Heilongjiang Province, Ning’an City, Bohai Town, Jingpo Lake National Scenic Area, 128°56′7.21″ N, 44°2′30.08″ E, alt. 379 m, 6 August 2025, light trap, leg. Z.C. Zhang.

**Distribution**: China (Heilongjiang, Jilin).


 


**Family Thremmatidae Martynov, 1935**

**Genus *Neophylax* McLachlan, 1871**

**Species *Neophylax ussuriensis* Martynov, 1914** (first record for China)

**Material examined**: 1 ♂, China, Heilongjiang Province, Ning’an City, Bohai Town, Jingpo Lake National Scenic Area, 128°56′7.21″ N, 44°2′30.08″ E, alt. 379 m, 6 August 2025, light trap, leg. Z.C. Zhang.

**Distribution**: China (Heilongjiang); South Korea [62].

### 3.2. Sequence Information

In terms of species richness, Hydropsychidae had the highest number of recorded species, with six species, followed by Leptoceridae with five species. Ecnomidae, Rhyacophilidae, Lepidostomatidae, and Molannidae each contained two species, whereas Limnephilidae, Philopotamidae, and Thremmatidae were each represented by one species.

At the species level, taxa with relatively high sample sizes were mainly concentrated in Leptoceridae and Hydropsychidae. For example, multiple individuals were obtained for *Ceraclea lobulata* and *Ceraclea albimacula*; within Hydropsychidae, *Macrostemum radiatum*, *Cheumatopsyche brevilineata*, and *Hydropsyche orientalis* were also represented by comparatively adequate numbers of individuals. In contrast, several species were represented by only a single individual, including *Arctopsyche palpata*, *Hydropsyche valvata*, *Oecetis brachyura*, *Ceraclea sibirica*, *Rhyacophila retracta*, and *Neophylax ussuriensis*.

In total, 105 COI barcode sequences were obtained in this study, representing 14 genera in 9 families of Trichoptera. Although chromatograms for some specimens of *Oecetis brachyura* and *Ceraclea sibirica* in Leptoceridae failed to meet the upload requirements of BOLD, their trace files could not be successfully submitted; the corresponding consensus sequences were still retained and included in all subsequent analyses.

The family-level distribution of sequences was uneven. Leptoceridae contained the largest number of sequences (35 sequences, 33.3%), followed by Hydropsychidae (28 sequences, 26.7%); together, these two families accounted for 60.0% of all sequences and constituted the core of the Jingpo Lake Trichoptera barcode dataset. The remaining taxa were represented by Ecnomidae (16 sequences, 15.2%), Rhyacophilidae (8 sequences, 7.6%), Lepidostomatidae (7 sequences, 6.7%), Limnephilidae (4 sequences, 3.8%), Molannidae (3 sequences, 2.9%), Philopotamidae (3 sequences, 2.9%), and Thremmatidae (1 sequence, 1.0%).

### 3.3. Phylogenetic Analysis

**Figure 3:**
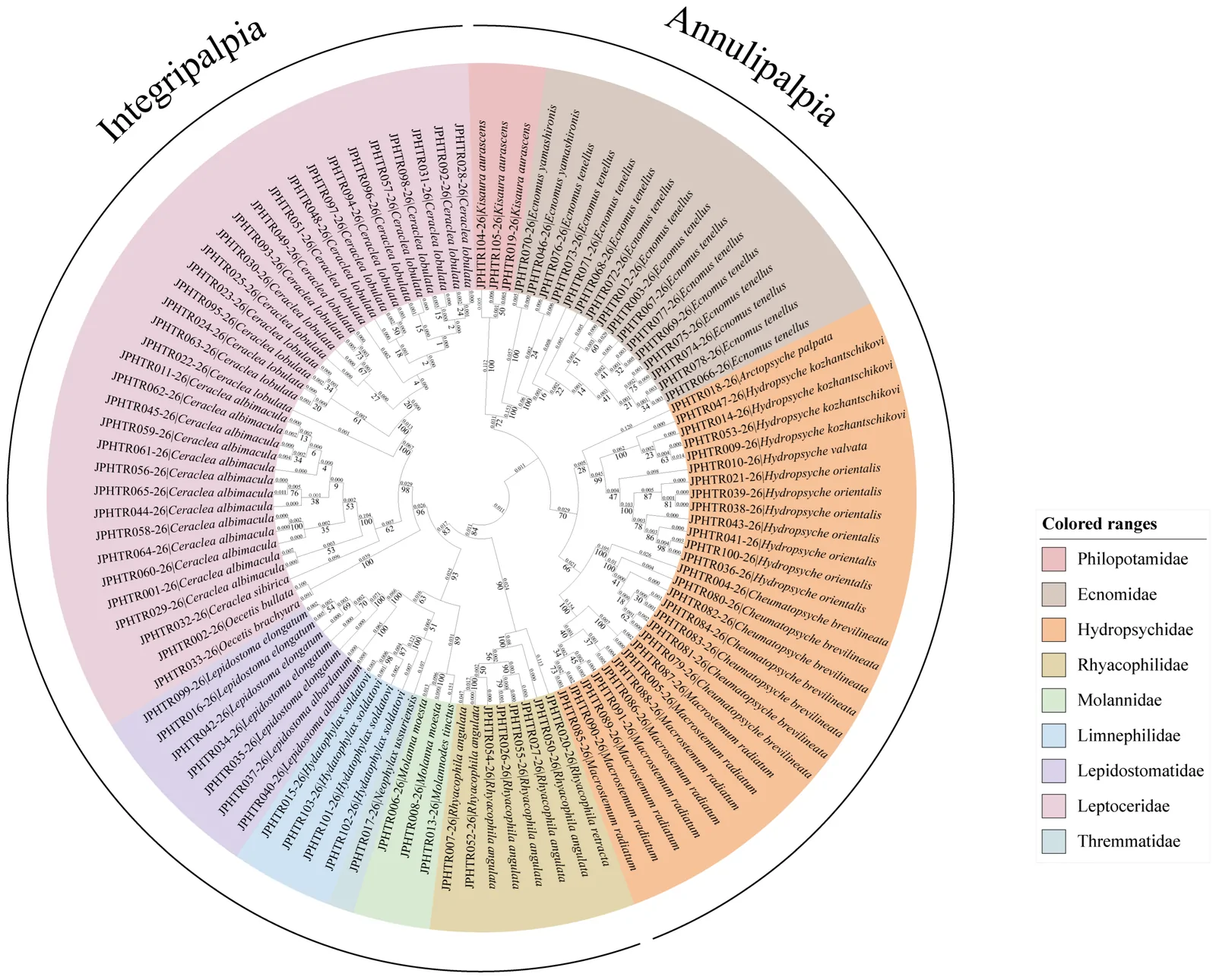
Neighbor-joining phylogenetic tree of Trichoptera from Jingpo Lake based on COI barcodes. The colored ring indicates different families within Trichoptera, as shown in the legend.

The NJ tree constructed from the 105 COI sequences showed an overall clear clustering pattern among taxonomic units (Figure 3), largely consistent with the results of morphological identification. At the family level, Leptoceridae, Hydropsychidae, Ecnomidae, Rhyacophilidae, Lepidostomatidae, Limnephilidae, Molannidae, Philopotamidae, and Thremmatidae each formed relatively independent branches, with no obvious cross-family mixing observed. This result indicates that COI barcodes effectively reflect genetic divergence among the major Trichoptera groups sampled in this study and can serve as an important complement to morphological identification.

Within families, most conspecific individuals clustered into terminal groups with short branches, suggesting low COI sequence divergence among individuals of the same species and relatively clear species boundaries. Within Leptoceridae, *Ceraclea albimacula*, *Ceraclea lobulata*, and *Ceraclea sibirica* each formed independent clusters, while *Oecetis bullata* and *Oecetis brachyura* were also clearly differentiated from the *Ceraclea* clade, indicating that both intergeneric and interspecific differences within this family were effectively captured by the COI barcode. Hydropsychidae exhibited a more complex branching pattern, with *Hydropsyche*, *Cheumatopsyche*, *Macrostemum*, and *Arctopsyche* each forming relatively distinct terminal groups. Species within *Hydropsyche* remained separable, suggesting that COI barcodes have good discriminatory power for closely related species in this family.

Within Ecnomidae, *Ecnomus tenellus* and *Ecnomus yamashironis* formed separate clusters; although these are closely related congeneric species, they were clearly separated in the NJ tree. In Lepidostomatidae, *Lepidostoma elongatum* and *Lepidostoma albardanum* also exhibited stable species-level clustering. For Molannidae, Philopotamidae, Limnephilidae, and Thremmatidae, which were represented by limited numbers of sequences, one or a few sequences constrained evaluation of the stability of intraspecific topology; nevertheless, these taxa remained clearly separated from other families in the tree, supporting the corresponding morphological identifications.

### 3.4. Genetic Differentiation and Barcode Gap Analysis

**Figure 4:**
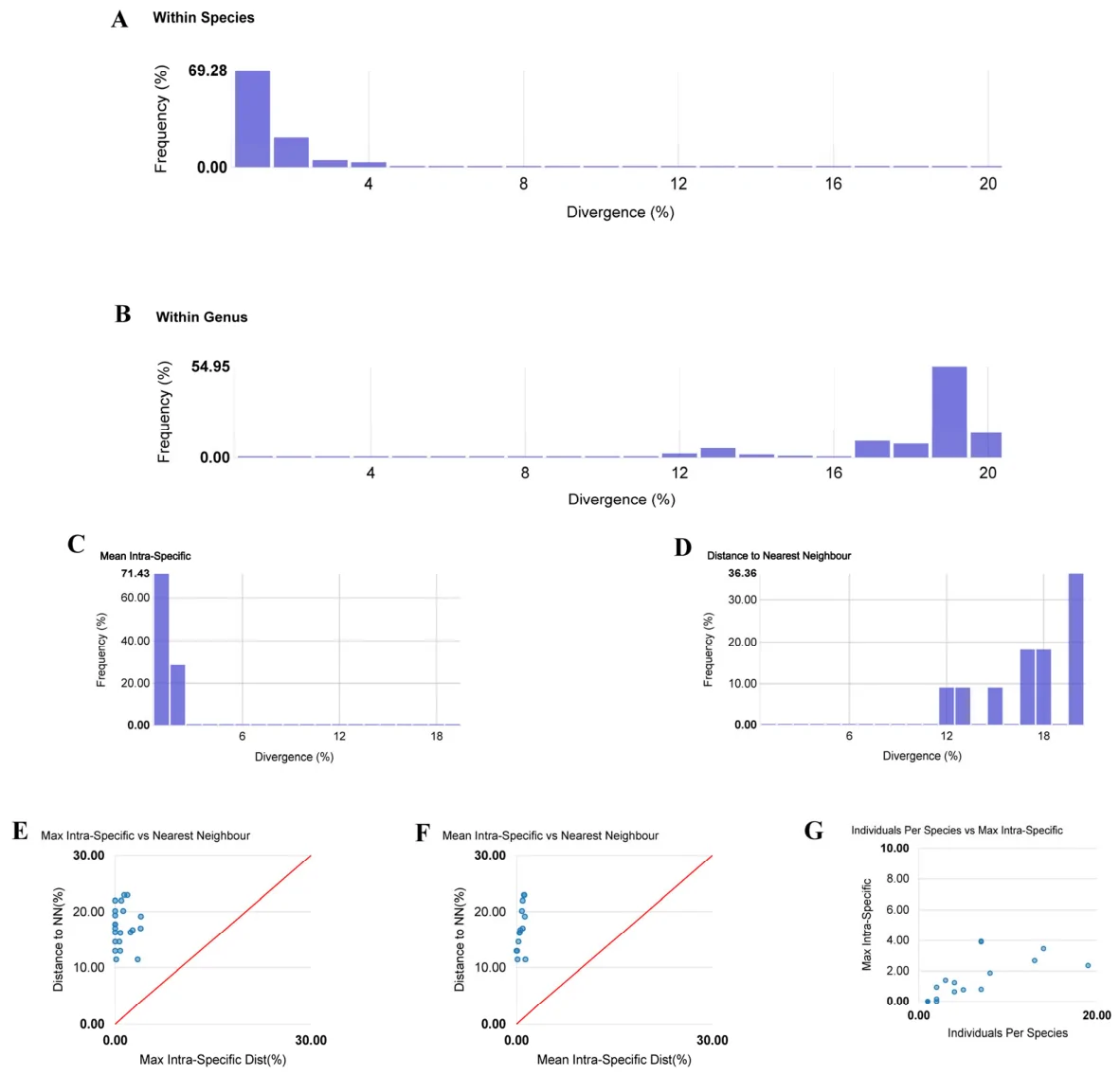
COI-based genetic divergence and barcode gap analysis of Trichoptera from Jingpo Lake. (**A**) Genetic distances within species; (**B**) genetic distances among species within the same genus; (**C**) mean intraspecific divergence for each species; (**D**) distances to the nearest neighboring species; (**E**) maximum intraspecific divergence plotted against nearest-neighbor distance; (**F**) mean intraspecific divergence plotted against nearest-neighbor distance; (**G**) relationship between the number of individuals sampled per species and maximum intraspecific divergence.

The dataset exhibited a distinct hierarchy of genetic distances across taxonomic levels. According to the BOLD Distance Summary results, 459 intraspecific pairwise comparisons were calculated for 97 valid sequences representing 14 species, with intraspecific K2P distances ranging from 0.00% to 3.94% and a mean of 0.78% (Figure 4A). After normalization for sample size, the mean intraspecific genetic distance was 0.69%, with a standard error of 0.03%, indicating that genetic differentiation among conspecific individuals was generally low and that this pattern was not strongly confounded by uneven sampling intensity among species. By contrast, 364 interspecific-within-genus comparisons were conducted for 78 sequences from 6 genera, with distances ranging from 11.45% to 21.34% and a mean of 17.80% (Figure 4B); 331 intrafamilial intergeneric comparisons were conducted for 66 sequences from 3 species-rich families, with distances ranging from 19.18% to 33.50% and a mean of 24.99%. Overall, genetic distances increased stepwise with rising taxonomic rank. The maximum intraspecific distance (3.94%) was markedly lower than the minimum intrageneric interspecific distance (11.45%), forming a clear intraspecific-interspecific distance discontinuity.

The distance distribution further showed that intraspecific genetic distances were concentrated mainly in the low-divergence range (Figure 4C), indicating high similarity among COI barcodes of most conspecific individuals. Intrageneric interspecific distances were mainly distributed in the higher divergence range of approximately 16–20% (Figure 4D), with little overlap with the intraspecific distance distribution. The normalized histogram further demonstrated that, after correcting for differences in sampling size, intraspecific distances remained concentrated at low values, whereas intrageneric interspecific distances remained high. These results indicate that the COI barcode can not only distinguish taxa at the family and genus levels but also provides stable molecular diagnostic boundaries at the species level in the present dataset.

Barcode gap analysis further supported the effectiveness of the COI barcode for species identification of the Trichoptera examined in this study. For all species, the maximum intraspecific genetic distance was lower than the minimum interspecific distance to the nearest-neighbor species (Figure 4E), and no absence of a barcode gap was detected. Overall, the mean of intraspecific distances across species was 0.44%, whereas the mean nearest-neighbor distance was 17.37%, with nearest-neighbor distances ranging from 11.45% to 22.93% (Figure 4F). The smallest nearest-neighbor distance occurred between *Ecnomus tenellus* and *Ecnomus yamashironis* (11.45%), but this distance was still substantially higher than the maximum intraspecific distances of the two species (3.45% and 0.16%, respectively), indicating that COI barcodes maintain a clear identification gap even between closely related congeneric species. A positive trend was observed between the maximum intraspecific distance and the number of individuals per species (Figure 4G).

Within Hydropsychidae, the genetic distance between *Hydropsyche kozhantschikovi* and its nearest-neighbor species *Hydropsyche valvata* was 14.65%, whereas the maximum intraspecific distance of *H. kozhantschikovi* was only 0.64%. The nearest-neighbor distance between *Hydropsyche orientalis* and *H. kozhantschikovi* was 16.25%, while the maximum intraspecific distance of *H. orientalis* was 0.80%. *Cheumatopsyche brevilineata* had a nearest-neighbor distance of 19.18% relative to *Hydropsyche kozhantschikovi*, and its maximum intraspecific distance reached 3.94%. The highest nearest-neighbor distance within the family was recorded between *Macrostemum radiatum* and *Cheumatopsyche brevilineata* (22.93%), with the former showing a maximum intraspecific distance of 1.86%. Within Leptoceridae, the distance between *Ceraclea albimacula* and its nearest-neighbor species *Ceraclea lobulata* was 16.73%, while the maximum intraspecific distance of *C. albimacula* was 2.67%. The nearest-neighbor distance between *C. lobulata* and *Ceraclea sibirica* was 16.32%, with a maximum intraspecific distance of 2.34% for *C. lobulata*. The nearest-neighbor distance between *Oecetis brachyura* and *Oecetis bullata* was 17.78%. These interspecific distances were all far higher than the corresponding intraspecific distances, indicating that COI barcodes provided stable species-identification signals even in taxa with relatively high species numbers and specimen counts. Within Lepidostomatidae, the nearest-neighbor distance between *Lepidostoma elongatum* and *Lepidostoma albardanum* was 12.98%, which was also substantially higher than the maximum intraspecific distance of *L. elongatum* (0.77%) and the intraspecific distance of *L. albardanum* (0.00%).

For taxa with relatively small sample sizes, including Molannidae, Philopotamidae, Limnephilidae, and Thremmatidae, the small number of specimens precluded a comprehensive assessment of intraspecific genetic variation. Nevertheless, these taxa still showed substantial genetic divergence from other groups and no unclear intraspecific divergence was observed. In the barcode gap scatterplot, all species were positioned above the equivalence line, indicating that nearest-neighbor interspecific distances were generally greater than maximum or mean intraspecific distances. Moreover, there was no clear positive correlation between the number of individuals per species and maximum intraspecific genetic distance.

### 3.5. Sampling Sufficiency and Accumulation Curves

The species accumulation curves showed that, based on 105 records, approximately 22 species-level taxonomic units and 14 genus-level taxonomic units were detected in the study area. The overall curve increased rapidly with increasing sample size during the early phase and then gradually decelerated, but it did not fully reach a horizontal plateau at the terminal portion. This pattern suggests that the present survey has covered the major genus-level composition of the Trichoptera assemblage in the Jingpo Lake study area, whereas species-level diversity may still be somewhat underestimated.

**Figure 5:**
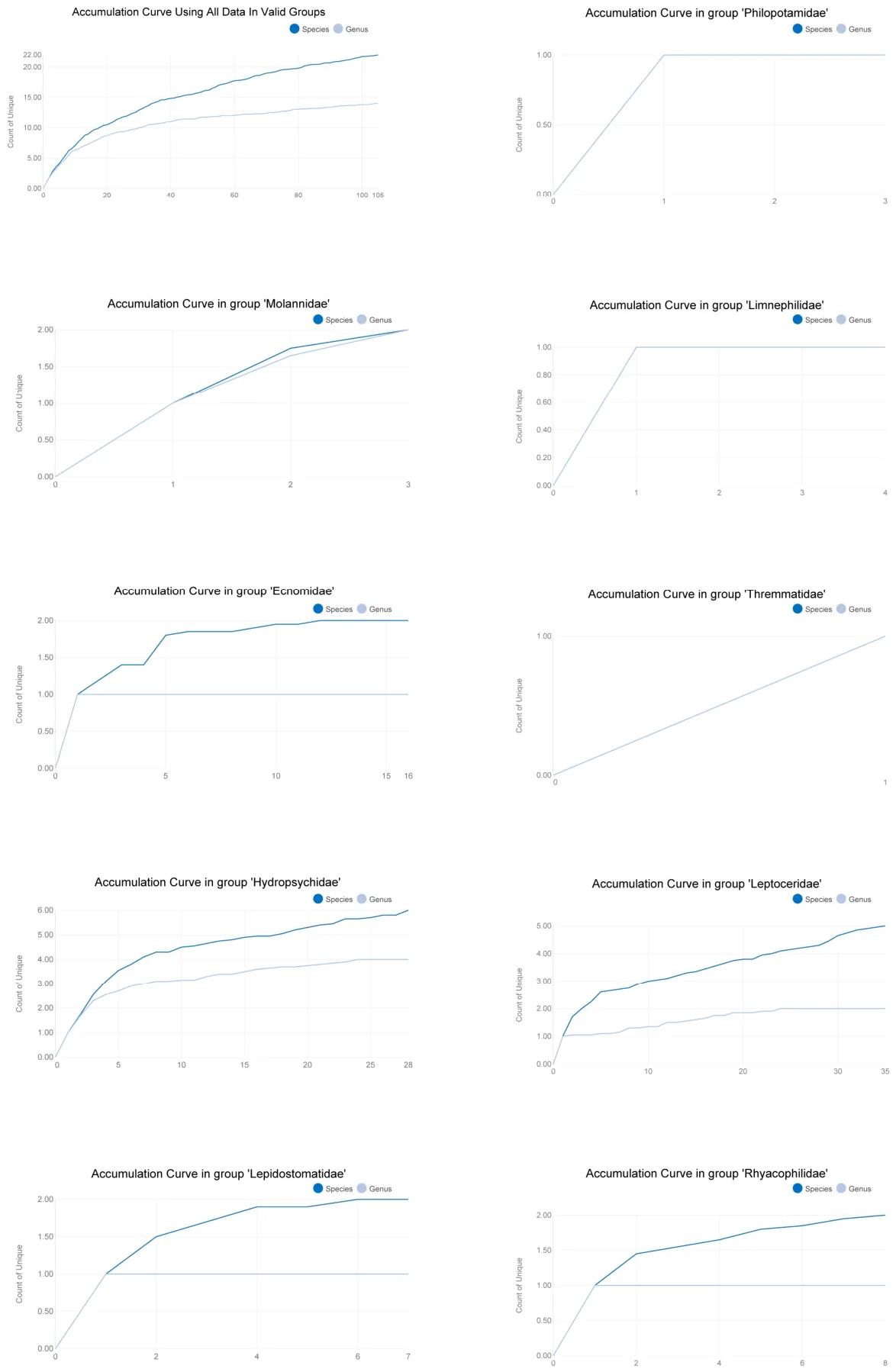
Accumulation curves for Trichoptera from Jingpo Lake. The curves illustrate the increase in cumulative species- and genus-level taxa as the number of COI sequences increases.

Accumulation trends differed markedly among families (Figure 5). Hydropsychidae accumulated approximately six species-level taxonomic units and was the most species-rich family in this study. Leptoceridae accumulated approximately five species and represented the family with the largest number of sequences and relatively high species richness. Ecnomidae, Lepidostomatidae, Rhyacophilidae, and Molannidae each contained approximately two detected species, whereas Philopotamidae, Limnephilidae, and Thremmatidae were each represented by only one detected species. This pattern is broadly consistent with the sequence composition and morphological identification results, indicating that the dominant families contributed not only a large proportion of individuals but also substantially to Trichoptera species diversity in the study area.

In terms of sampling sufficiency, the genus-level curve of Ecnomidae approached a plateau relatively early, while its species-level curve gradually approached two species in the later phase, suggesting relatively stable genus-level coverage for this family in the present sampling. The curves for Lepidostomatidae and Rhyacophilidae also gradually approached a plateau, implying that the collected samples may largely represent their main species composition during the survey period. The curves for Philopotamidae and Limnephilidae reached plateaus relatively quickly because of their low species numbers; however, this plateau mainly reflects the detection outcome within the current sample set and does not necessarily indicate that the true regional diversity has been exhaustively sampled.

By contrast, the species-level curves of Hydropsychidae and Leptoceridae continued to rise slowly in the later phase. Hydropsychidae, in particular, continued to show species accumulation even at a relatively high sample size, suggesting that additional species in this family may remain insufficiently sampled. In Leptoceridae, the genus-level curve tended to stabilize, whereas the species-level curve still showed a slight increase, indicating that its genus-level composition may have been largely captured but that intrageneric species diversity may still be underestimated. The curves for Molannidae and Thremmatidae should not be overinterpreted: records for Molannidae were limited and the curve remained sensitive to the addition of new individuals, while Thremmatidae was represented by only one sequence and its curve mainly reflects insufficient sampling.

## 4. Discussion

### 4.1. Trichoptera Diversity and Biogeographic Significance in Northern China

Previous taxonomic studies of Trichoptera in China have mainly focused on southern China, particularly in regions with complex topography, well-developed river systems, and high habitat heterogeneity, such as Yunnan, Guangxi, Guangdong, Fujian, Hainan, Guizhou, and Sichuan. These areas are characterized by abundant mountain streams, subtropical forests, and diverse freshwater habitats, which provide suitable conditions for the diversification of caddisflies. Previous studies have shown that China harbors a high diversity of Trichoptera, and new species and new records continue to be discovered. For example, the amended checklist of Chinese Trichoptera by Yang et al. [37] recorded 1267 described species belonging to 116 genera and 30 families, and indicated that numerous newly described and newly recorded species had been added to the Chinese fauna within a relatively short period. Therefore, continued regional taxonomic surveys remain essential for improving our understanding of Trichoptera diversity in China.

In contrast, the caddisfly fauna of northern China has received comparatively less attention, and the available taxonomic information remains limited. For instance, Morse et al. [19], in their study of the Trichoptera of Liaoning Province, noted that Liaoning was one of the least known regions for caddisflies in China. The previously recorded species number was relatively low, whereas a single new collection yielded several new provincial records, new records for Palaearctic China, and new records for China. This suggests that the apparent low diversity of Trichoptera in northern China may largely reflect insufficient sampling and limited taxonomic investigation rather than true species paucity.

Northern China is located in a transitional area between the Oriental and Palaearctic regions and is therefore of particular biogeographic significance. The caddisfly fauna of this region may contain elements from both zoogeographic regions and regional taxa adapted to northern mountain streams and related freshwater habitats. Accordingly, northern China should not be regarded simply as a region with poor Trichoptera diversity, but rather as an important area with considerable potential for further faunal discovery. In the present study, 285 specimens of Trichoptera were collected from northern China, representing 22 species, among which four species are newly recorded from China. These findings suggest that additional undocumented species, new distributional records, and even potentially undescribed taxa may still be discovered in northern China. Further investigations will contribute to a more comprehensive understanding of species diversity, distribution patterns, and faunal composition of Trichoptera in northern China, and will provide important baseline data for improving the taxonomy and biogeographic knowledge of Chinese Trichoptera.

### 4.2. COI Barcode for Species Identification

Based on 105 Trichoptera COI barcode sequences collected from Jingpo Lake National Scenic Area, this study integrated morphological identification, NJ clustering, genetic distance estimation, barcode gap analysis, and species accumulation curves to comprehensively evaluate the performance of molecular identification and the coverage of diversity in the study area. The results showed that COI barcodes effectively distinguished the major families, genera, and species examined in this study. Most conspecific individuals clustered into compact branches, each family formed relatively independent clusters in the NJ tree, mean intraspecific genetic distances were markedly lower than interspecific-within-genus and intergeneric-within-family distances, and all species showed clear barcode gaps. Collectively, these findings demonstrate that the COI barcode is suitable for rapid identification of adult Trichoptera in the Jingpo Lake region and can provide a reliable molecular basis for freshwater insect diversity surveys, refinement of regional species inventories, and subsequent ecological monitoring.

Larval stages of Trichoptera predominantly inhabit freshwater environments and are sensitive to flow velocity, substrate type, input of organic particles, and water-quality conditions; consequently, they are widely used in assessments of freshwater ecosystem health [19]. In this study, Hydropsychidae and Leptoceridae exhibited the highest species numbers or sequence numbers, while also including several low-abundance or occasionally encountered taxa, indicating that Jingpo Lake and its surrounding streams and lakeshore habitats can support diverse Trichoptera assemblages. Larvae of Hydropsychidae are typical net-spinning caddisflies and are commonly associated with running-water habitats. They usually construct fixed silken retreats on the surfaces of stones or other submerged substrates, with capture or filtering nets at or near the retreat openings to collect suspended organic particles from the current [63,64]. This adaptation to lotic microhabitats may explain their relatively high diversity in streams and inflowing water systems within the study area. Larvae of Leptoceridae often construct cases from plant debris or mineral particles and may occupy still waters, slow-flowing waters, and microhabitats rich in aquatic vegetation [65]. Their high sequence number and incompletely saturated accumulation curve suggest that additional, insufficiently sampled taxa may occur in lakeshore zones, wetland margins, and slow-flowing habitats.

The genetic distance results provide quantitative support for species identification in this study. Across all samples, the mean intraspecific K2P distance was only 0.78%, and the maximum intraspecific distance was 3.94%, whereas the minimum interspecific-within-genus distance reached 11.45%, producing a clear discontinuity between the two levels. This pattern of low intraspecific and high interspecific divergence is a key condition for the effective application of DNA barcoding and also indicates good agreement between morphological identification and COI molecular evidence in the present samples. For species-rich groups such as Hydropsychidae, even when intraspecific divergence exceeded 2% in some species, the nearest-neighbor interspecific distance remained far greater than the maximum intraspecific distance and therefore did not cause overlap in identification boundaries. Such relatively high intraspecific divergence may be associated with geographic isolation, local population structure, historical expansion, or unrecognized lineage divergence [66,67,68,69], and may also be influenced by retention of mitochondrial haplotypes, mitochondrial capture, or nuclear mitochondrial copies [70]. Because this study did not incorporate nuclear genes or population genetic data, these mechanisms require further testing. Within the current dataset, the more conservative interpretation is that these species exhibit a certain degree of intraspecific lineage divergence, but this divergence has not compromised the effectiveness of COI barcoding for identification. Taken together, the intraspecific distances, nearest-neighbor distances, and NJ clustering results indicate that COI barcodes can reliably distinguish the trichopteran species collected in Jingpo Lake.

The species accumulation curves further revealed differences in sampling sufficiency among families. The curves for Ecnomidae, Lepidostomatidae, Philopotamidae, and Limnephilidae approached plateaus, suggesting that the present survey may have covered these groups relatively adequately. For Hydropsychidae, Leptoceridae, and Rhyacophilidae, genus-level curves were relatively stable whereas species-level curves continued to rise slowly, indicating that additional species records may be obtained by expanding sampling sites, seasons, and microhabitat types in future work. For Molannidae and Thremmatidae, unsaturated curves more likely reflect limited sample size rather than directly demonstrating exceptionally high true diversity. In particular, Thremmatidae was represented by only one sequence, and any inference regarding its richness should therefore be treated with caution. These results suggest that future surveys should prioritize increased sampling intensity for Hydropsychidae, Leptoceridae, Molannidae, and Thremmatidae, with repeated collections across seasons, water-body types, and microhabitats to more accurately estimate the true species richness of Trichoptera in the Jingpo Lake region.

This study has practical implications for regional biodiversity surveys and freshwater ecological monitoring. Jingpo Lake and its inflowing streams form a relatively complex lake-stream ecosystem, in which Trichoptera larvae play important roles in organic matter decomposition, particle filtration, and energy transfer within aquatic food webs [19]. Establishing a local COI barcode dataset can improve the efficiency of identifying Trichoptera from adults, larvae, and environmental samples, while reducing misidentifications caused by incomplete life stages, damaged specimens, or morphological similarity among closely related species when identification relies solely on morphology. As the local reference library continues to expand, these data can also support DNA metabarcoding and environmental DNA monitoring, providing higher-resolution species-level evidence for assessing the ecological quality of lakes and streams [71].

## 5. Conclusions

This study established a localized COI barcode reference dataset for Trichoptera in the Jingpo Lake region based on 105 representative adult specimens selected from 285 collected individuals and covering all species recorded during the survey. The dataset comprised 22 species, 14 genera, and 9 families, thereby providing important baseline molecular information for a freshwater insect group that has been insufficiently documented in Northeast China. The NJ tree, K2P genetic distance analysis, and barcode gap analysis consistently supported the effectiveness of COI-5P in distinguishing the sampled Trichoptera species, with low intraspecific divergence, high interspecific divergence, and clear barcode gaps across all examined species. These results demonstrate strong concordance between morphological identification and molecular evidence in the present dataset. The accumulation curves further suggest that the current survey captured the principal genus-level composition of the assemblage, but that species-level diversity, especially in Hydropsychidae, Leptoceridae, Molannidae, and Thremmatidae, may remain incompletely sampled. Therefore, future work should expand sampling across seasons, habitats, and life stages, and should integrate larval morphology, ecological information, nuclear markers, and metabarcoding data. Overall, the barcode library generated here provides a practical foundation for species identification, regional biodiversity assessment, environmental DNA-based monitoring, and long-term conservation management of Jingpo Lake and related lake-stream ecosystems.

## Data Availability

The data presented in this study are openly available in the Barcode of Life Data System (BOLD; https://v4.boldsystems.org, accessed on 30 June 2026) under the dataset “Barcoding Study of Trichoptera in Jingpo Lake, Heilongjiang Province, China (JPHTR)”.
